# Draft Genome Sequences of Two *Clostridium botulinum* Group II (Nonproteolytic) Type B Strains (DB-2 and KAPB-3)

**DOI:** 10.1128/genomeA.01111-14

**Published:** 2014-11-06

**Authors:** Nicholas Petronella, Robyn Kenwell, Franco Pagotto, Arthur W. Pightling

**Affiliations:** a Biostatistics and Modelling Division, Bureau of Food Surveillance and Science Integration, Food Directorate, Health Products and Food Branch, Health Canada, Ottawa, Ontario, Canada; b Microbiology Research Division, Bureau of Microbial Hazards, Food Directorate, Health Products and Food Branch, Health Canada, Ottawa, Ontario, Canada

## Abstract

*Clostridium botulinum* is important for food safety and studies of neurotoxins associated with human botulism. We present the draft genome sequences of two strains belonging to group II type B: one collected from Pacific Ocean sediments (DB-2) and another obtained during a botulism outbreak (KAPB-3).

## GENOME ANNOUNCEMENT

*Clostridium botulinum* is a Gram-positive, anaerobic bacterium that is present naturally in soils and sediments around the world (1). *C. botulinum* produces a highly potent neurotoxin that may cause botulism when ingested (2). Botulism is characterized by a wide range of symptoms from vomiting and gastrointestinal distress to cranial nerve palsies that may progress to complete flaccid paralysis and death due to respiratory failure (3).

*C. botulinum* strains are organized into four metabolically and genetically distinct groups (I–IV) (4). Groups I and II are associated with human botulism (5). Group II is nonproteolytic and organized into three serotypes (A, B, or F) that are distinguished by the botulinum neurotoxin produced (5). Group II strains ferment a number of sugars, form spores with moderate heat resistance, are often linked with outbreaks of food-borne botulism involving fish and meat (6), and are a concern in the safe production of chilled ready meals (7).

Despite the importance of group II type B strains to food safety and studies of neurotoxin gene clusters (8), comprehensive sequencing of their genomes has only recently been performed. Here, we present the high-quality draft genome sequences of *C. botulinum* strain DB-2, obtained from sediments in 1968 from the Pacific Ocean, and strain KAPB-3, linked to a 1981 botulism outbreak caused by kapchunka (dried salted whitefish) in the United States.

Short-read sequence data were generated for these two genomes by preparing paired-end libraries with the Nextera XT DNA sample preparation kit (Illumina, San Diego, CA) and sequencing the libraries on an Illumina MiSeq benchtop sequencer for 500 cycles. The number of reads generated were 2,074,424 for DB-2 and 1,991,848 for KAPB-3. Error correction was performed with BayesHammer (9), and reads were assembled *de novo* into high-quality draft genomes with SPAdes version 3.1.1 (10), utilizing the MismatchCorrector tool. In total, the DB-2 assembly yielded 150 nonoverlapping contiguous sequences with 108-fold coverage, 27.18% GC content, and a combined length of 3,915,341 bases. The KAPB-3 assembly generated 128 nonoverlapping contiguous sequences with 112-fold coverage, 27.17% GC content, and a total length of 3,871,084 bp. Gene predictions and annotations were performed with the National Center for Biotechnology Information (NCBI) Prokaryotic Genome Annotation Pipeline (PGAP) (11). For DB-2 a total of 3,570 genes were identified, including 3,384 protein-coding regions, 54 pseudogenes, 50 rRNA sequences, and 81 transfer RNAs. For KAPB-3 a total of 3,509 genes were identified, including 3,325 protein-coding regions, 56 pseudogenes, 49 rRNA sequences, and 78 transfer RNAs.

### Nucleotide sequence accession numbers.

These whole-genome shotgun projects have been deposited at DDBJ/EMBL/GenBank under accession numbers JQOJ00000000 (DB-2) and JQOK00000000 (KAPB-3). The versions described in this paper are the first versions, JQOJ01000000 and JQQK01000000.

## References

[1] Dodds KL 1993 *Clostridium botulinum* in the environment, p 105–120 *In* Hauschild HW Dodds KL (ed), Clostridium botulinum: ecology and control in foods. Marcel Dekker, New York, NY.

[2] Austin JW 2001 Clostridium botulinum, p 329–349 *In* Doyle MP Beuchat LR Montville TJ (ed), Food microbiology: fundamentals and frontiers, 2nd ed. ASM Press, Washington, DC.

[3] Shapiro RL Hatheway C Swerdlow DL 1998 Botulism in the United States: a clinical and epidemiologic review. Ann. Intern. Med. 129:221–228. 10.7326/0003-4819-129-3-199808010-00011. 9696731

[4] Peck MW 2009 Biology and genomic analysis of *Clostridium botulinum*. Adv. Microb. Physiol. 55:183–265, 320. 10.1016/S0065-2911(09)05503-9. 19573697

[5] Stringer S Carter A Webb M Wachnicka E Crossman L Sebaihia M Peck M 2013 Genomic and physiological variability within group II (non-proteolytic) *Clostridium botulinum*. BMC Genomics 14:333. 10.1186/1471-2164-14-333. 23679073 PMC3672017

[6] Peck MW Stringer SC 2005 The safety of pasteurised in-pack chilled meat products with respect to the foodborne botulism hazard. Meat Sci. 70:461–475. 10.1016/j.meatsci.2004.07.019. 22063745

[7] Peck MW Goodburn KE Betts RP Stringer SC 2008 Assessment of the potential for growth and neurotoxin formation by non-proteolytic *Clostridium botulinum* in short shelf-life commercial foods designed to be stored chilled. Trends Food Sci. Technol. 19:207–216. 10.1016/j.tifs.2007.12.006.

[8] Carter AT Stringer SC Webb MD Peck MW 2013 The type F6 neurotoxin gene cluster locus of group II *Clostridium botulinum* has evolved by successive disruption of two different ancestral precursors. Genome Biol Evol. 5:1032–1037. 10.1093/gbe/evt068. 23645598 PMC3673618

[9] Nikolenko SI Korobeynikov AI Alekseyev MA 2013 BayesHammer: Bayesian clustering for error correction in single-cell sequencing. BMC Genomics 14(Suppl 1):S7. 10.1186/1471-2164-14-S1-S7. 23368723 PMC3549815

[10] Bankevich A Nurk S Antipov D Gurevich AA Dvorkin M Kulikov AS Lesin VM Nikolenko SI Pham S Prjibelski AD Pyshkin AV Sirotkin AV Vyahhi N Tesler G Alekseyev MA Pevzner PA 2012 SPAdes: a new genome assembly algorithm and its applications to single-cell sequencing. J. Comput. Biol. 19:455–477. 10.1089/cmb.2012.0021. 22506599 PMC3342519

[11] Angiuoli SV Gussman A Klimke W Cochrane G Field D Garrity G Kodira CD Kyrpides N Madupu R Markowitz V Tatusova T Thomson N White O 2008 Toward an online repository of standard operating procedures (SOPs) for (meta)genomic annotation. Omics 12:137–141. 10.1089/omi.2008.0017. 18416670 PMC3196215

